# Identifying Key Metabolites in South African Medicinal Plants Using Dual Electrospray Ionization Metabolomics

**DOI:** 10.3390/plants15020232

**Published:** 2026-01-12

**Authors:** Mmamudi Anna Makhafola, Clarissa Marcelle Naidoo, Chikwelu Lawrence Obi, Benson Chuks Iweriedor, Oyinlola Oluwunmi Olaokun, Earl Prinsloo, Muhammad Sulaiman Zubair, Nqobile Monate Mkolo

**Affiliations:** 1 Department of Biology and Environmental Sciences, Sefako Makgatho Health Sciences University, Pretoria 0204, South Africa; 201008578@swave.smu.ac.za (M.A.M.); clarissa.naidoo@smu.ac.za (C.M.N.); lawrence.obi@smu.ac.za (C.L.O.); benson.iweriebor@smu.ac.za (B.C.I.); oyinlola.olaokun@smu.ac.za (O.O.O.); 2 Department of Biotechnology, Rhodes University, Makhanda 6140, South Africa; e.prinsloo@ru.ac.za; 3 Department of Pharmacy, University of Tadulako, Palu 94118, Indonesia; sulaimanzubair@untad.ac.id

**Keywords:** untargeted metabolomics, dual electrospray ionization mode, positive ionization mode, negative ionization mode, *Lippia javanica*, *Acorus calamus*

## Abstract

Despite growing interest in South African medicinal plants, advanced metabolomic workflows that integrate positive (ESI+) and negative (ESI−) ionization modes in UPLC-MS/MS remain sparsely applied to South African flora, and especially to *Acorus calamus* and *Lippia javanica* species. Herein, application of a dual-polarity (positive (ESI+) and negative (ESI−) ionization modes) using an untargeted UPLC–MS/MS workflow, integrated with HEK293T cytotoxicity screening, to map their metabolomes, and rank potential signature metabolites for targeted antiviral follow-up. SwissADME supported in silico drug-likeness. Neither plant extract was cytotoxic across the concentration range, with absorbance-based cell viability of 73.82% for *L. javanica* and 77.23% for *A. calamus* at 250 µg/mL, and fluorescence-based cell viability ≥59.87% and ≥55.89%, respectively. Dual-polarity expanded coverage with ESI− yielded 312 features, compared with 225 with ESI+, consistent with the predominance of acidic phenolics in plant species. Unsupervised and supervised models segregated the plant species (PCA PC1/PC2 variance: ESI+ 89.4%/3.0%; ESI− 93.5%/1.8%; R^2^X(cum) = 0.799). Differential analysis identified 118 significant features in ESI+ with 80 up-regulated, 38 down-regulated, and 139 in ESI− with 96 up-regulated, 43 down-regulated. The ESI− showed the wider dynamic range. Chemotypes enriched among significant metabolites include flavonols of 3-O-methylkaempferol, apigenin, and conjugates of Pollenin A, iridoid glycosides of oleoside, forsythoside B, and jasmonate-pathway oxylipins of 7-epi-12-hydroxyjasmonic acid and its glucoside. These also include caryoptosidic acid and catechin-7-glucoside, which are ionized in both modes, pinning the increase in biomarker robustness. In conclusion, a dual-mode UPLC–MS/MS approach, integrated with cytotoxicity exploration, delivers a complementary metabolome coverage and a safety awareness for shortlisting of potential signature metabolites from *L. javanica* and *A. calamus*. Moreover, in vitro inhibition of SARS-CoV-2 papain-like protease (PL^pro^) by these plants links chemical signatures to antiviral relevance. Shortlisted significant metabolites that demonstrated favorable drug-likeness include flavonol scaffolds of 3-O-methylkaempferol, Pollenin A, and jasmonate-pathway derivatives of 7-epi-12-hydroxyjasmonic acid. Moreover, the dual ionization mode may eliminate ionization bias, broaden metabolome coverage, and yield a mechanism-ready shortlist of metabolites from South African medicinal plants for downstream antiviral investigation.

## 1. Introduction

People have turned to plants as their first line of defense against illness for centuries. Many of today’s medicines, from aspirin to artemisinin, trace their roots back to natural products, with more than half of all approved drugs being plant-derived or inspired by them [1]. This long-standing success underscores why scientists continue to study medicinal plants, particularly in times when new and re-emerging diseases remain a global concern. Even with vaccines and antiviral drugs, the coronavirus pandemic has demonstrated how vulnerable we are to viral mutations and to unequal access to healthcare resources worldwide [2]. Exploring traditional remedies that are already widely used and culturally accepted may therefore provide affordable and effective alternatives or complements to modern treatments.

In South Africa and across the continent, *Lippia javanica* and *Acorus calamus* are two plants with a long history of traditional use for colds, fever, and other respiratory conditions [3,4]. Notably, the introduced form of *A. calamus* growing in South Africa has been reported to be the triploid cytotype [5,6], while *L. javanica* ploidy in South Africa is not well established [7], which is particularly relevant to mention because different cytotypes are recognized to produce distinct chemical profiles [8]. Known locally as “fever tea” or “sweet flag,” these plants are not only part of cultural heritage but also possess antimicrobial, anti-inflammatory, and immune-supporting properties [9,10]. Moreover, decoctions or infusions of *A. calamus* roots and *L. javanica* leaves are commonly used by local communities as home remedies for flu-like symptoms, implying potential antiviral and immunomodulatory activity [9,10]. Yet, while people have trusted them for generations, we still do not fully understand which chemical compounds are responsible for their effects or how safe they are at different doses. Oblivious to such knowledge, it is challenging to validate their use scientifically or to further advance these plants toward reliable therapeutic solutions. To date, comprehensive metabolomic profiling of these South African medicinal plant species using dual-polarity electrospray ionization in both positive and negative modes remains unreported, mainly, despite the region’s rich phytochemical diversity [11]. This gap underscores the novelty of applying a dual-mode UPLC-MS/MS workflow to these species and supports the methodological contribution of this study. The comparative analysis of *L. javanica* and *A. calamus* was intentional and hypothesis-driven, based on shared ethnomedicinal importance rather than taxonomic proximity. Comparative LC-MS metabolomics can also be applied to diverse plant species to establish comprehensive chemical fingerprints, identify chemotype-specific versus shared scaffolds, and prioritize candidate constituents for downstream bioactivity testing. In the context of botanical natural products, such workflows are recommended because they enable systematic characterization of chemically complex matrices and support rational compound prioritization rather than anecdotal selection [12,13,14,15,16].

Modern metabolomic approaches offer a path to bridging this knowledge gap. Coupled with ultra-performance liquid chromatography-mass spectrometry (UPLC-MS), investigators can detect thousands of molecules in plant matrices, unraveling both primary metabolites, such as nucleotides and amino acids, and secondary metabolites, including phenolic acids and flavonoids [17,18]. Running these analyses in both positive and negative electrospray ionization (ESI) modes can enhance metabolome coverage and limit the loss of significant compounds that may emerge only under certain conditions [18,19]. Integrating this approach with multivariate statistical modeling and in silico pharmacokinetic prediction platforms such as SwissADME [20] permits not only the identification of potentially significant metabolites but also the prediction of drug-like metabolites.

This study, therefore, aimed to comprehensively characterize the metabolomes of *L. javanica* and *A. calamus* using dual electrospray UPLC-MS/MS, integrate in silico drug-likeness and ADME predictions with HEK293T cytotoxicity profiling to establish preliminary safety margins, and generate a mechanism-driven shortlist of candidate metabolites with potential relevance for the development of new antiviral agents against respiratory viral infections. Accordingly, this present study integrates untargeted metabolomics with targeted in vitro antiviral screening to link chemical profiling and early-stage antiviral discovery.

## 2. Results

### 2.1. Resazurin Cell Viability Assay

**Figure 1:**
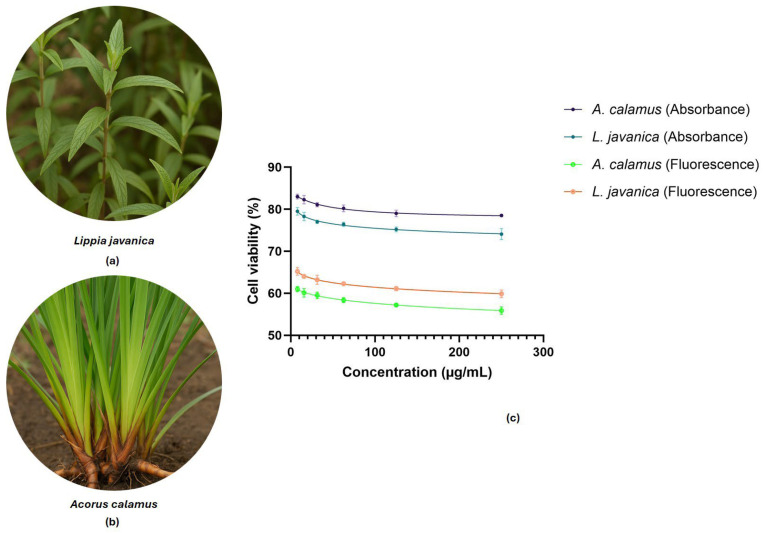
The HEK293T cell viability was measured using resazurin reduction assay and fluorescence-based detection after treatment with (**a**) *L. javanica* and (**b**) *A. calamus* extracts at different concentrations ranging from 250 to 7.81 µg/mL. (**c**) Data are shown as mean ± SD (*n* = 3) with error bars.

Cytotoxicity of methanolic extracts of *L. javanica* and *A. calamus* was evaluated against HEK293T cells utilizing the resazurin reduction assay within a gradient of concentration (7.81–250 µg/mL). Both plant extracts showed elevated cell viability across all concentrations tested, indicating a favorable safety profile (Figure 1). At the highest concentration tested of 250 µg/mL, HEK293T cell viability was 73.82% for *L. javanica* and 77.23% for *A. calamus* after quantification using absorbance of resorufin at 570 nm. Even at a reduced concentration of 7.81 µg/mL, both plant extracts maintained high viability, with no evidence of dose-dependent toxicity in the cells. Fluorescence-based detection further confirmed these findings, with HEK293T viability remaining above 59.87% for *L. javanica* and 55.89% for *A. calamus* across all tested concentrations. Test for differences across concentrations was conducted, and statistically significant variation in cell viability values was observed (*L. javanica* absorbance: F = 12.21, *p* < 0.001; *A. calamus* absorbance: F = 7.30, *p* = 0.002; *L. javanica* fluorescence: F = 14.34, *p* < 0.001; *A. calamus* fluorescence: F = 16.52, *p* < 0.001).

### 2.2. Metabolic Profiling

**Figure 2:**
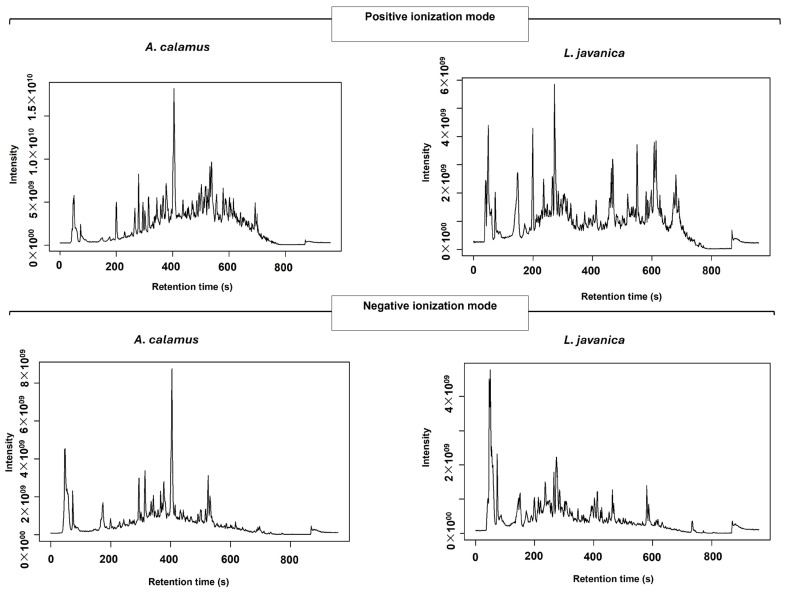
Typical example of base peak intensity obtained from samples of *A. calamus* and *L. javanica* in ESI+ (positive mode) and ESI− (negative mode) using UPLC-MS/MS analyses.

The chromatograms of *A. calamus* and *L. javanica* were examined in two ionization modes, namely, ESI+ (positive mode) and ESI− (negative mode). These modes aid in identifying various bioactive compounds in these medicinal plants. Following data processing and annotation, a total of 225 features were detected under ESI+ mode and 312 features under ESI− mode, across both *L. javanica* and *A. calamus*. Supporting Information for Table S1 presents a comprehensive chemical profile of these features, along with additional details. The base peak intensity chromatograms of *L. javanica* and *A. calamus* samples in ESI+ and ESI− modes are provided in Figure 2.

### 2.3. Multivariate Data Analysis

**Figure 3:**
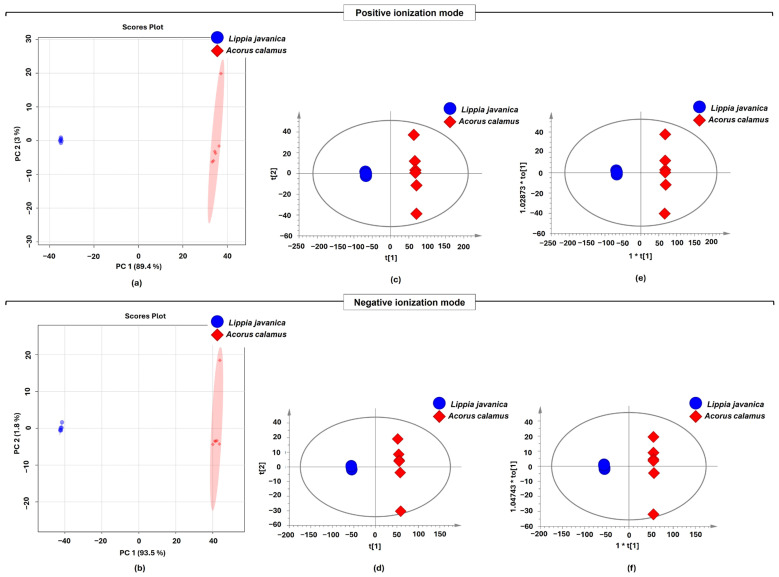
Multivariate data analysis: The scores scatter plot of PCA (**a**,**b**), scores scatter plot of PLS-DA (**c**,**d**)**,** and OPLS-DA (**e**,**f**) models depicting a cluster of *L. javanica* and *A. calamus* metabolites. Key: The asterisk (*) presenting the score notation denotes the component index. t[1] and t[2] denote the first and second predictive (class-related) score components, respectively, whereas to[1] represents the first orthogonal (class-unrelated) component.

The data for the identified signature compounds, including retention times (RT), *m*/*z* values, human metabolome database identification (HMDB_ID), compound names, formulas, VIP values, *t*-test results, and fold change (FC), are presented in Table 1 and Table 2. The ESI+ and ESI− peaks were combined and then loaded into SIMCA-P for multivariate statistical analysis. To study global metabolic changes, principal component analysis (PCA) was applied to all metabolites obtained in both ion modalities (Figure 3). PCA score plots signify the distribution of biological samples based on the collective metabolomic profiles. As shown in the PCA score scatter plots, an overview of metabolites in the dataset was provided, with a clear grouping pattern between the two groups of *L. javanica* and *A. calamus*. The two principal components (PCs) explained by the samples examined in positive ionization mode accounted for 89.4% of the variance in PC1 and 3% in PC2. In contrast, negative ionization mode accounted for 93.5% in PC1 and 1.8% in PC2 (Figure 3). R^2^ (cum) is a metric used to assess the quality of a PCA model, with values close to 1.0 indicating high fitness and good predictive ability. In this study, R^2^X (cum) is 0.799, indicating that the developed PCA model exhibits sufficient fitness and predictive ability (Table 3).

Partial Least Squares Discriminant Analysis (PLS-DA) and Orthogonal Partial Least Squares Discriminant Analysis (OPLS-DA) were used in both positive and negative ionization modes to maximize class discrimination between *L. javanica* and *A. calamus*. The PLS-DA and OPLS-DA score plots (t2 vs. t1) showed clear separation between the two groups of *L. javanica* and *A. calamus* (Figure 3). The PLS-DA and OPLS-DA score plots’ principal component 1 (PC1) accounted for most of the variance in both modes, with 89.4% and 80.1% in the positive and negative modes, respectively. Principal component 2 (PC2) accounted for 2% and 4.8% of the variance in the positive and negative modes, respectively (Figure 3). Particularly in PC1, the negative ionization mode showed a larger variance difference (4.1%, 7.8%) than the negative ionization modes (Figure 3). Table 3 presents the supervised PLS-DA and OPLS-DA models, which offered more robust separation, model fit, and predictability across both ionization modes.

Together, the analyses follow a sequential framework in which unsupervised PCA offers a global overview of variance, supervised PLS-DA and OPLS-DA classify discriminative features, and univariate plots summarize the fold-change directionality and statistical relevance of the top VIP-ranked metabolites for biological interpretation.

### 2.4. Univariate Data Analysis

**Figure 4:**
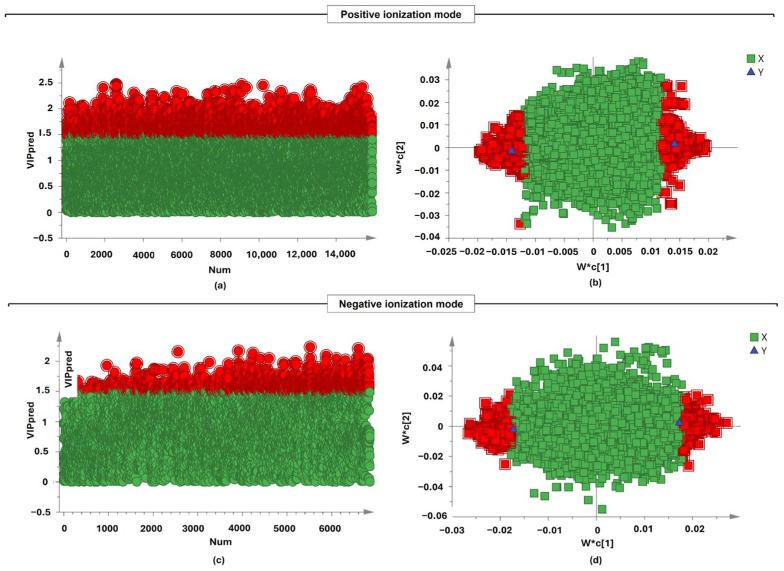
Illustration of relative fold-change directionality and statistical relevance of selected metabolites. The distribution of VIP values (VIP > 1.5) (**a**,**c**) and the loading plot of the PLS-DA model (**b**,**d**) for positive ionization mode and negative ionization mode. The *L. javanica* and *A. calamus* metabolites with red color represent significant metabolites, and the metabolites with green color represent non-significant compounds. Key: W∗c[1] and W∗c[2] represents the first and second predictive loading-correlation vectors.

The selection criteria for significant metabolites were based on VIP > 1.5, FC > 2.0, and adjusted *p*-value < 0.05, with class annotations presented in Table 1 and Table 2. In ESI+, discriminant metabolites were dominated by flavonoids and flavonoid conjugates, with contributions from phenolic acid conjugates, jasmonate-derived oxylipins, glycosylated carbohydrates, and nucleoside derivatives. While ESI- highlighted phenylethanoid glycosides, phenolic conjugates, iridoid glycosides, triterpenoid derivatives, and lipid-related metabolites. The metabolites (ions) that differed significantly between the *L. javanica* and *A. calamus* groups were filtered using VIP values (VIP > 1.5) from multivariate analysis of the full curated feature sets reported in Supplementary Table S1. Figure 4 illustrates the relative importance, fold-change directionality, and statistical significance of the selected metabolites for complementing multivariate score plots. The OPLS-DA loading plot is shown in Figure 4, and the metabolites with a red box are labeled as significant compounds. In the positive ionization mode, many features exceeded the VIP > 1.5 threshold, as evidenced by the dense accumulation of red markers above this threshold (Figure 4). This indicates a high concentration of discriminative metabolites in the positive ionization mode, with VIP values reaching approximately 2.6. In comparison, the negative mode had a reduced overall density of high VIP values (Figure 4). Although a similar number of features exceeded the VIP > 1.5 criterion, their distribution was slightly more dispersed, with no peaks exceeding 2.0. This suggests a less pronounced but still significant set of discriminant traits in the negative ionization mode. Overall, both ionization modes successfully identified multiple key metabolites; however, the positive ionization mode yielded more features and greater discriminative power, as indicated by VIP scores.

**Figure 5:**
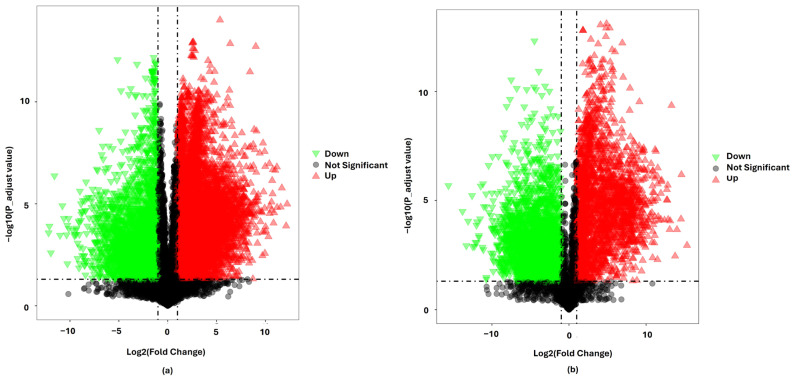
Volcano plot for *L. javanica* and *A. calamus* groups. The range of Y > 1.30 and X > 1 was a significant increase; The range of Y > 1.30 and X < −1 was a significant decrease. Key: (**a**) positive ionization mode; (**b**) negative ionization mode.

To study the variations in metabolite expression between *L. javanica* and *A. calamus* groups, volcano plots were created for both positive and negative ionization modes (Figure 5). The plots show the log_2_ fold change (x-axis) vs. the −log_10_ adjusted *p*-value (*y*-axis), with thresholds set at log_2_FC > 1 or < −1 and −log_10_(*p*-adj) > 1.30 for statistical significance.

A large number of metabolites were intensely altered while using the positive ionization mode (Figure 5). A dense cluster of up-regulated metabolites was found with a fold change range of log_2_(FC) of +4.488 to +11.679 (~22-fold to ~3300-fold up-regulation), while down-regulated metabolites ranged from log_2_(FC) of −4.970 to −8.772 (~31-fold to ~430-fold down-regulation). The distribution of −log_10_(*p*-adj) values showed substantial statistical significance for these differentially expressed metabolites. A total of 118 metabolites were significant, with VIP values greater than 1.5; 80 were up-regulated and 38 down-regulated. Among the up-regulated metabolites (top 15), which were significantly increased (Y > 1.30 and X > 1), include 7-Epi-12-hydroxyjasmonic acid, Isopropyl beta-glucoside, Pollenin A, 3′-C-Ethynylcytidine, 6b-Hydroxymethandienone, 2-trans-O-Feruloylglucaric acid, Quercetin 3-O-glucuronide, Persiconin, Neolicuroside, Apigenin 7-O-diglucuronide, Hesperidin methylchalcone, Tetramethylquercetin 3-rutinoside, Hetastarch, and Leonoside A. Among the down-regulated (top 15), which were significantly decreased (Y > 1.30 and X < −1) include 5-Methylcytosine, 5-Isothiocyanatoindane, 2′,3′-Didehydro-2′,3′-dideoxycytidine, Pantothenic acid, 2-Hydroxyacorenone, Linamarin, 1-Hydroxyacorenone, 2′,3′-Dideoxyadenosine, Glycyl-Tryptophan, 5-Methyldeoxycytidine, Thromboxane B3, 7a,12a-Dihydroxy-cholestene-3-one, DG(20:4-2OH/0:0/2:0), Procyanidin and Marmesin rutinoside. The distinct separation of highly up- and down-regulated metabolites demonstrates the remarkable selective power of positive-mode ionization in detecting physiologically important alterations.

In contrast, among the 139 significant metabolites identified in negative ion mode, 96 were up-regulated, and 43 were down-regulated (Figure 5). Metabolites were up-regulated with a fold change range of log_2_ (FC) of +6.9661 to +14.0073 (~125 to ~16,467 fold up-regulation) and down-regulated with a fold change range of log_2_ (FC) of −6.9664 to −11.9818 (~125× to ~4045 fold down-regulation). Beyond a ±10 log_2_ fold change, indicating more dramatic alterations in levels during this mode. The top 15 up-regulated metabolites that were significantly increased (Y > 1.30 and X > 1), include 3-O-Methylkaempferol, DHAP(6:0), 7-Epi-12-hydroxyjasmonic acid glucoside, MG(13:0/0:0/0:0), Oleoside, Sweroside, 3-Methoxy-4-hydroxyphenylglycol glucuronide, Flurbiprofen glucuronide, Secogalioside, Isoyatein, Todatriol glucoside, 4-phenylbutanic acid-O-sulfate, Forsythoside B, Apigenin 7-glucuronide and Betanidin 5-[E-feruloyl-(->5)-apiosyl-(1->2)-glucoside]. The top 15 down-regulated metabolites that were significantly decreased (Y > 1.30 and X < −1) includes 9,11-Tetradecadienal, Caffeic acid 3-O-sulfate, Fukiic acid, 7(14)-Bisabolene-2,3,10,11-tetrol, 6-Hydroxyenterolactone, Tirandamycin B, Catechin 7-glucoside, MG(22:5-O(16,17)/0:0/0:0), Ononin, 6-beta-Glucopyranosyl-4′,5-dihydroxy-3′,7-dimethoxyflavone, Theasapogenol E, Corosin, Kaempferol 3-O-alpha-(3-trans-p-coumaroyl-rhamnopyranoside), Nemorubicin and PA(15:0/20:4-OH(15S)).

The number of significant metabolites remained large, but the variance in −log_10_(*p*-adj) values was higher than in the positive ionization mode. Notably, the negative ionization mode identified metabolites with larger fold changes and a wider dynamic range, demonstrating its ability to capture highly responsive metabolic characteristics.

### 2.5. Shared Metabolites Identified in Both Ionization Modes

**Figure 6:**
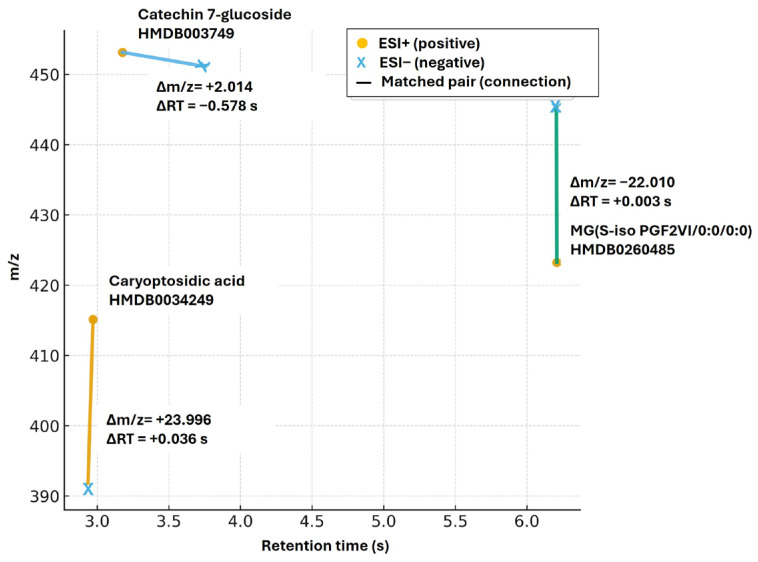
Retention time (min) vs. *m*/*z* map showing shared significant metabolites of the positive and negative ionization modes.

Caryoptosidic acid, Catechin 7-glucoside, and MG (5-iso PGF2VI/0:0/0:0) were identified as significant common metabolites to both the positive and negative ionization modes (Figure 6). Contrarily, some significant metabolites exclusively appeared in the positive ionization mode, including 1-Hydroxyacorenone, 1 (2H)-Pentalenone, 1,3-Hexadien-3-amine, 1-O-Caffeoyl-beta-glucose, and 1-(Ribofuranosyl)indoline. Contrariwise, several significant metabolites exclusively appeared in the negative ionization mode, including (1-O-Feruloyl-beta-glucose, 1-O-Sinapoyl-beta-glucose 3-Nitrobenzoyl) alanine, 1-Naphthol, and 1-O-Caffeoyl-(b-glucose 6-O-sulfate).

### 2.6. Pharmacokinetic Properties and Drug-Likeness Evaluation

**Figure 7:**
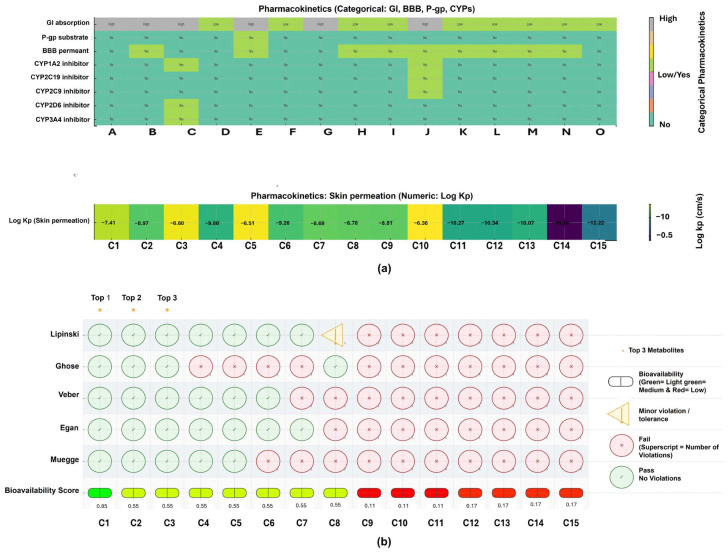
(**a**) Pharmacokinetic properties and (**b**) drug-likeness evaluation of significant metabolites from positive ionization mode (ESI+). **Key: C1/A:** 7-Epi-12-hydroxyjasmonic acid; **C2/C:** Pollenin A; **C3/E:** 6b-Hydroxymethandienone; **C4/B:** Isopropyl beta-glucoside; **C5/G:** 2′,3′-Didehydro-2′,3′-dideoxycytidine; **C6/D:** 3′-C-Ethynylcytidine; **C7/J:** DG(20:4-2OH/0:0/2:0); **C8/I:** Persiconin; **C9/F:** 2-trans-O-Feruloylglucaric acid; **C10/H:** Quercetin 3-O-glucuronide; **C11/K:** Apigenin 7-O-diglucuronide; **C12/L:** Hesperidin methylchalcone; **C13/M:** Tetramethylquercetin 3-rutinoside; **C14/N:** Hetastarch; **C15/O:** Leonoside A. **GI:** Gastrointestinal; **BBB:** blood–brain barrier; **P-gp:** P-glycoprotein; **CYP:** Cytochrome P450 enzymes. The superscript number next to each red symbol indicates the number of violated criteria within that rule set (e.g., Lipinski, Ghose, Veber, Egan, and Muegge).

**Figure 8:**
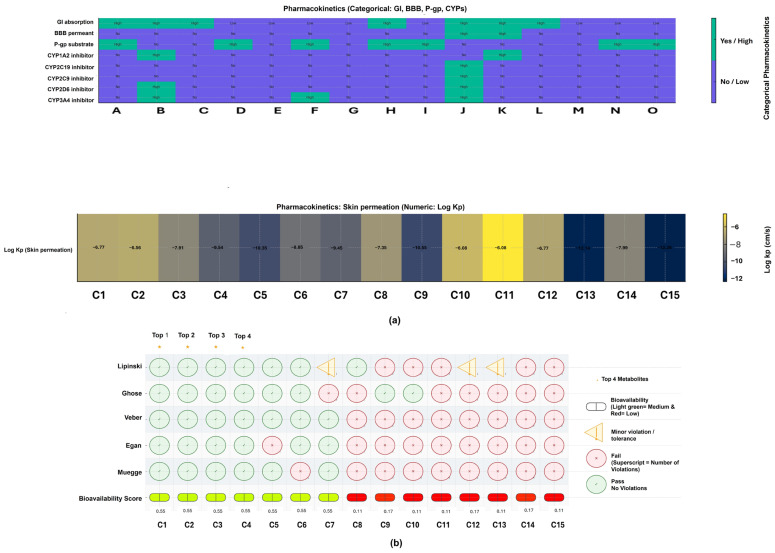
(**a**) Pharmacokinetic properties and (**b**) drug-likeness evaluation of significant metabolites from negative ionization mode (ESI−). **Key: C1/B**: 3-O-Methylkaempferol; **C2/C**: DHAP(6:0); C3/J: Isoyatein; C4/L: 4-phenylbutanic acid-O-sulfate; **C5/H**: Flurbiprofen glucuronide; **C6/K**: 9,11-Tetradecadienal; **C7/A**: Theasapogenol E; **C8/D**: 7-Epi-12-hydroxyjasmonic acid glucoside; **C9/F**: 6-beta-Glucopyranosyl-4′,5-dihydroxy-3′,7-dimethoxyflavone; **C10/N**: Apigenin 7-glucuronide; **C11/E**: Oleoside; **C12/G**: 3-Methoxy-4-hydroxyphenylglycol glucuronide; **C13/I**: Secogalioside; **C14/M**: Forsythoside B; C15/O: Betanidin 5-[E-feruloyl-(->5)-apiosyl-(1->2)-glucoside]. **GI**: Gastrointestinal; **BBB**: blood–brain barrier; **P-gp**: P-glycoprotein; **CYP**: Cytochrome P450 enzymes. The superscript number next to each red symbol indicates the number of violated criteria within that rule set (e.g., Lipinski, Ghose, Veber, Egan, and Muegge).

The SwissADME database was used to predict the water solubility, lipophilicity, pharmacokinetics, and drug-likeness of 15 top signature metabolites (Supplementary Material Table S2, Figure 7 and Figure 8). Metabolites in the positive ionization mode ranged from small molecules, such as 7-Epi-12-hydroxyjasmonic acid (226.27 g/mol), to large glycosides, such as Leonoside A (770.73 g/mol) (Figure 7). More minor metabolites, including 7-Epi-12-hydroxyjasmonic acid and Pollenin A (Apigenin), met Lipinski’s rule of five with no violations. Lipophilicity ranged from hydrophilic substances such as isopropyl beta-glucoside (Log Po/w = −1.11) to lipophilic aglycones such as 6b-Hydroxymethandienone (Log Po/w = 2.76), thereby influencing solubility and membrane permeability. Water solubility ranged greatly from highly soluble 3′-C-Ethynylcytidine (3.48 × 10^2^ mg/mL) to poorly soluble Pollenin A (Apigenin) (8.46 × 10^−2^ mg/mL). Smaller compounds showed greater absorption, whereas large glycoside molecules, such as Apigenin 7-O-diglucuronide (283.34 Å^2^), exhibited low permeability, as indicated by polar surface area (TPSA) results. Based on the pharmacokinetic study, small, less-polar metabolites, including 7-Epi-12-hydroxyjasmonic acid and 6b-Hydroxymethandienone, exhibit high gastrointestinal absorption, with 6b-Hydroxymethandienone also crossing the blood–brain barrier. While these molecules exhibited strong drug-likeness and good bioavailability, larger glycosides showed poor absorption and low scores. Medicinal chemistry warnings identified hazards on compounds such as Pollenin A, displaying PAINS alerts and probable CYP inhibition, implying toxicity and drug–drug interactions. Nonetheless, 7-Epi-12-hydroxyjasmonic acid, Pollenin A, and 6b-Hydroxymethandienone exhibit favorable drug-liking profiles relative to the other top 15 signature metabolites (Figure 7).

The SwissADME results for the 15 significant metabolites in the negative ionization mode (ESI−) provide important insights into their potential as drug candidates, based on their physicochemical properties, lipophilicity, water solubility, pharmacokinetic behavior, drug-likeness, and medicinal chemistry. Furthermore, based on the results obtained, a diverse range of structures was observed, from small molecules such as 4-phenylbutanic acid-O-sulfate (244 g/mol) to large glycosides, such as betanidin 5-[E-feruloyl-(->5)-apiosyl-(1->2)-glucoside] (859 g/mol). This size variation significantly affects their drug-like properties, with smaller compounds generally complying more closely with Lipinski’s rule of five. The lipophilicity results show a wide spectrum, from highly hydrophilic glycosides, for example, Betanidin 5-[E-feruloyl-(->5)-apiosyl-(1->2)-glucoside] (Log Po/w −3.78), to more lipophilic compounds such as isoyatein (Log Po/w 3.34), which influences both their solubility and membrane permeability. The water solubility of compounds varies widely: some glycosides, such as 7-Epi-12-hydroxyjasmonic acid glucoside, exhibit high solubility, whereas others, such as Isoyatein, have more limited solubility. These variations are not supported by polar surface area (TPSA) values, which indicate that smaller molecules, such as isoyatein (TPSA 72 Å^2^), have superior absorption potential, whereas 7-Epi-12-hydroxyjasmonic acid glycosides with large TPSA (153.75 Å^2^) exhibit poor membrane permeability. The observed pharmacokinetic behavior, in which smaller, less polar molecules exhibit better gastrointestinal absorption and, in certain situations, blood–brain barrier penetration, is closely correlated with these physicochemical qualities. Medicinal chemistry alerts indicate that substances such as forsythoside B exhibit catechol signals that may contribute to potential toxicity. Nonetheless, 3-O-Methylkaempferol, DHAP (6:0), Isoyatein, and 4-phenylbutanic acid-O-sulfate are the molecules that exhibit favorable profiles in terms of drug likeness compared to other molecules of the top 15 signature metabolites (Figure 8).

## 3. Discussion

The study compared metabolomic profiles of *L. javanica* and *A. calamus* using a dual ESI mode UPLC-MS/MS metabolomics technique. However, the safety profiles of both plant extracts were validated by cytotoxicity assays. Cell viability was demonstrated by the resazurin assay at all tested concentrations (7.81–250 µg/mL). Slight divergence between fluorescence- and absorbance-based resazurin readouts reflects the higher sensitivity of fluorescence to early metabolic redox perturbations compared to absorbance [21,22]. In this study, the concordant retention of ≥55% viability across both resazurin detection modes at 250 µg/mL supports the interpretation that the extracts exert, at most, marginal cytostatic effects under screening conditions, consistent with accepted thresholds for early-stage exploratory natural-product bioassays [23]. The bioactive chemicals in these extracts may operate on specific targets without causing general cellular harm; their lack of cytotoxicity is a crucial factor in considering them for further therapeutic development [24]. Thus, untargeted metabolomic analysis was further achieved to reveal signature metabolites of *L. javanica* and *A. calamus* using a dual ionization mode. Although *L. javanica* and *A. calamus* are botanically distinct, their comparative analysis was driven by their shared ethnomedicinal use for respiratory conditions. Untargeted metabolomics permits broader coverage of specialized metabolites, and multivariate chemometric analyses facilitate the identification of conserved and divergent chemotypes among species. This comparative framework has been applied to natural product research to prioritize and contextualize bioactive compounds across diverse biological samples, thereby highlighting species-specific metabolic signatures relevant to their reported therapeutic uses [12,13,14,15,16]. In this study, the combination of positive and negative electrospray ionization modes was critical for obtaining a more complete metabolic profiling of both medicinal plant species. The incorporation of dual electrospray ionization modes substantially improved metabolome coverage and interpretative depth in this study. Negative ionization specifically captured phenolic and acidic metabolites, comprising oxylipins and glycosylated flavonoids, where positive ionization enhanced the detection of less acidic compounds and neutral scaffolds. The complementary nature of ESI+ and ESI− consequently enabled a more comprehensive characterization of the chemical space of *L. javanica* and *A. calamus*, reducing ionization bias and strengthening confidence in metabolite prioritization [25,26,27]. This complete profiling is essential not only for precise comparative analysis but also for improving quality control and pharmacological assessment of medicinal plants used in traditional medicine, such as *L. javanica* and *A. calamus*. The incorporation of ESI+ and ESI− modes improves quality control (QC) in untargeted metabolomics by reducing ionization bias and enabling complementary detection of acidic and basic metabolites, thereby minimizing false negatives from single-mode analyses [25]. Cross-mode verification enhances annotation confidence and reproducibility by enabling the consistent discovery of significant metabolites across ESI+ and ESI− datasets [26]. In addition, observing constancy of ion ratios and retention-time alignment across modes provides internal evidence of instrument performance and data integrity, supporting robust comparative analyses [27]. According to Cech and Enke [28] and Cole [29], when ESI/MS is coupled to liquid chromatography (LC), the positive-ion mode is typically selected because more compounds are expected to ionize in this mode. Although the positive-ion mode is preferred, the negative-ion mode has the advantage of reduced background noise [28,29]. Currently, there is a scarcity of research and guidance on which mode to use when a substance ionizes in both modes. One key component in interpreting the ESI process is the solvent composition, which is typically defined by the initial composition due to the difficulty of measuring the actual composition in the column. However, it has been demonstrated that the solvent pH, organic solvent concentration, and droplet size vary along the column [30,31]. Moreover, isomers and derivatives of asarone (*β*- and *α*-asarone) are also not likely detected due to low ionization in UPLC-ESI-MS/MS systems [32], the methoxylated aromatic structure of asarone might lead to inefficient ionization under the particular source conditions (example, ESI), or the formation of stable adducts (example, sodium adducts), can reduce fragment ion production [32,33].

According to the UPLC-MS/MS results, the negative-ionization mode with 312 retained features exhibited a greater number of features than the positive-ionization mode with 225 retained features, consistent with the nature of compounds typically found in plants. In this study, ESI− facilitated superior structural elucidation of glycosides and phenolic acids, whereas ESI+ improved annotation of flavonoids and alkaloids. According to Commisso et al. [17], compounds belonging to chemical classes such as flavonoids, organic acids, and phenolic acids, all of which are prevalent in medicinal plants, are among the acidic substances that the negative ionization mode is extremely sensitive to. The positive ionization mode, by contrast, was most effective at ionizing neutral and basic compounds, such as alkaloids, amino acids, and specific lipids. The positive ionization mode’s higher base peak intensity for *A. calamus* highlights a higher abundance of compounds that ionize effectively as positive ions, such as metabolites that contain nitrogen. Moreover, compounds that can easily accept a proton (H^+^) to become positively charged ([M + H]^+^ ions) are best ionized by positive mode [34]. These bioactive constituents, which typically include amines, alkaloids, and some glycosides, are often basic. On the other hand, compounds that may readily donate a proton to become negatively charged ([M − H]^−^ ions) are the ideal candidates for negative ionization mode. These are often acidic bioactive constituents, such as organic acids, phenolics, and phosphorylated or sulfated molecules [35]. Compounds such as 1-hydroxyacorenone, 1(2H)-pentalenone, 1,3-Hexadien-3-amine, 1-O-caffeoyl-β-glucose, and 1-(ribofuranosyl)indoline easily accept protons (protonated) and are examples of metabolites that only ionize in positive ionization mode. Conversely, highly acidic compounds, like 1-O-feruloyl-β-glucose and 1-O-sinapoyl-β-glucose, only ionize in negative ionization mode because their carboxylic or sulfate groups readily deprotonate or donate a proton [29].

Some compounds, however, contain both acidic and basic functional groups and can therefore ionize in both acidic and basic environments. In this study, caryoptosidic acid, Catechin 7-glucoside, and MG (5-iso PGF2VI/0:0/0:0) were identified as common, significant metabolites in both positive and negative ionization modes, indicating their versatility in ionization. Compounds such as caryoptosidic acid (an Iridoid glycoside), commonly found in plant species, contain both hydroxyl (-OH) and carboxylic acid (-COOH) [36,37], enabling detection in both modes. The flavonoid glucoside catechin 7-glucoside likewise exhibits dual ionization: its sugar moiety can be protonated in the positive ionization mode, whereas its phenolic -OH groups favor negative ionization [28,38]. Moreover, MG (5-iso PGF2VI/0:0/0:0), a lipid derivative of an isoprostane, contains hydroxyl and carboxylic acid groups that, depending on structure, enable detection in both ionization modes [39]. Given the potential detection of 5-Fluoromethylornithine, which could indicate a novel fluorinated metabolite, this finding would be unusual because natural enzymatic fluorination is exceptionally rare and restricted to a few microorganisms, for example, *Streptomyces cattleya* [40,41]. Thus, establishing this as new biochemical information would require comprehensive structural and biosynthetic evidence to exclude analytical or database artifacts.

Multivariate statistical analyses using PCA, PLS-DA, and OPLS-DA revealed distinct differences between metabolites in *L. javanica* and *A. calamus* across both ionization modalities. The substantial cumulative R^2^X and Q^2^ values for the models suggest strong and consistent separation, highlighting the two species’ different chemical identities. In particular, the PCA model’s R^2^X(cum) value was 0.799, which is considered robust for exploratory analysis in untargeted metabolomics and supports the reliability of the observed sample clustering [42]. Moreover, univariate data analysis of metabolites (ions) that differed significantly between the *L. javanica* and *A. calamus* groups was performed. Revealing a total of 118 metabolites, with 80 up-regulated and 38 down-regulated for positive ionization mode. In contrast, among the 139 significant metabolites identified in negative ionization modes, 96 were up-regulated, and 43 were down-regulated. Utilizing positive ionization mode, a dense cluster of up-regulated metabolites was found with a fold change range of log_2_ (FC) of ~22-fold to ~3300-fold up-regulation, while down-regulated metabolites ranged from log_2_ (FC) of ~31-fold to ~430-fold. While the negative ionization mode metabolites were up-regulated with a fold change range of log_2_ (FC) of ~125 to ~16,467 fold, and down-regulated with a fold change range of log_2_ (FC) of ~125× to ~4045 fold. These results suggest that a large number of metabolites were markedly altered under positive-ionization conditions.

In the positive ionization mode, the revealed top 15 significant metabolites are 7-Epi-12-hydroxyjasmonic acid, Isopropyl beta-glucoside, Pollenin A, 3′-C-Ethynylcytidine, 6b-Hydroxymethandienone, 2-trans-O-Feruloylglucaric acid, 2′,3′-Didehydro-2′,3′-dideoxycytidine, Quercetin 3-O-glucuronide, Persiconin, DG(20:4-2OH/0:0/2:0), Apigenin 7-O-diglucuronide, Hesperidin methylchalcone, Tetramethylquercetin 3-rutinoside, Hetastarch, and Leonoside A. These significant metabolites belong to different chemical classes, including flavonoids and derivatives, phenylethanoid glycosides, phenolic acid derivatives, oxylipins, steroids, nucleosides, glycosides, and polysaccharides, with flavonoids and derivatives comprising the largest proportion. Among these significant metabolites, 7-Epi-12-hydroxyjasmonic acid, Pollenin A, and 6b-Hydroxymethandienone exhibit favorable drug-likeness profiles and are all up-regulated.

Amongst the discriminative metabolites, oxylipins linked with the jasmonate pathway appeared as important contributors to interspecies differentiation. Jasmonate-derived oxylipins are central regulators of plant defense and stress responses and have been progressively associated with the modulation of host–pathogen interactions and immune-relevant signaling pathways. Their abundance difference implies species-specific activation of defense-related metabolic pathways, which may highlight the reported bioactivities of these plants in traditional medicinal contexts [43,44]. A plant-derived metabolite of 7-Epi-12-hydroxyjasmonic acid is a jasmonate derivative, part of the oxylipin pathway in higher plants [45]. Jasmonates are lipid-derived phytohormones that regulate plant defense, stress adaptation, and senescence. They can be isolated from plants, for example, *Jasminum* spp. and other jasmonate-producing species [46]. While their antiviral effects are documented in plants, for instance, resistance to Rice ragged stunt virus, limited evidence exists yet for direct antiviral properties in human systems. However, this metabolite has been studied for antioxidant, anti-inflammatory, and anticancer effects, and its similarity to eicosanoids or prostaglandins suggests the potential to modulate signaling pathways in human cells [46,47,48].

Flavonoids and iridoid glycosides are further substantiated as the chemical signatures distinguishing the two species. These metabolite classes are commonly recognized for their antiviral, immunomodulatory, and anti-inflammatory properties, with reported mechanisms comprising interference with viral proteases, regulation of inflammatory signaling cascades, and modulation of host redox balance. The enrichment of these scaffolds among top VIP-ranked metabolites provides mechanistic plausibility, linking the observed metabolomic differences to the ethnomedicinal use of both plants and highlighting candidate compounds for downstream functional validation [49,50]. Pollenin A, also known as the flavonol herbacetin in chemical databases, can be derived from medicinal plants that are used for making tea [49]. A wider variety of pharmacological effects, such as anticancer and antidiabetic properties, as well as strong enzyme-inhibitory activity, particularly against certain cytochrome P450 (CYP) and other metabolic enzymes, have also been demonstrated in vitro and in animal models [50]. Crucially, however, most of the available information is preclinical, and there is a conspicuous lack of clinical data from human trials to support these potential therapeutic uses or health benefits. Moreover, there is limited knowledge of its potential as an antiviral agent. Unlike natural plant flavonoids or jasmonates, 6β-Hydroxymethandienone has no pharmaceutical, antiviral, or nutritional uses. Nonetheless, flavonoids such as kaempferol, apigenin, and quercetin have shown promising antiviral activity against SARS-CoV-2 by targeting key viral enzymes and receptors [51,52]. Based on this study, these metabolites can inhibit viral entry and replication by binding to the host ACE2 receptor and the viral main protease (3CLpro). Given that kaempferol and apigenin were detected in these plants, their flavonoid profile may similarly interfere with viral protease activity, supporting their potential as plant-derived antiviral agents. In this study, we adopted a pre-mechanistic metabolomic prioritization approach rather than a direct evaluation of antiviral efficacy. Untargeted dual-mode LC-MS metabolomics was used to systematically identify and prioritize metabolite classes with known or plausible roles in antiviral activity, thereby narrowing the chemical space for subsequent functional validation. To strengthen the translational relevance of the metabolomic findings, *L. javanica* and *A. calamus* extracts were then evaluated by applying an in vitro SARS-CoV-2 papain-like protease (PL^pro^) inhibition assay (Supplementary Figure S1). PL^pro^ is a well-recognized antiviral target that plays a significant role in viral polyprotein processing and immune evasion, and its inhibition represents a proven strategy for suppressing coronavirus replication [53]. Importantly, metabolite classes highlighted by the multivariate analysis, comprising iridoid glycosides, flavonoids, and oxylipin-related compounds, have been previously reported to inhibit viral proteases, modulate host inflammatory signaling, or interrupt virus–host interactions [54,55,56,57]. The convergence of metabolomic prioritization with PL^pro^ inhibition data, therefore, offers a biologically meaningful link between the chemical signatures identified in this study and their possible relevance to the development of antiviral agents targeting respiratory viral infections.

The negative ionization mode, on the other hand, detected the top 15 significant metabolites of Theasapogenol E, 3-O-Methylkaempferol, DHAP(6:0), 7-Epi-12-hydroxyjasmonic acid glucoside, Oleoside, 6-beta-Glucopyranosyl-4′,5-dihydroxy-3′,7-dimethoxyflavone 3-Methoxy-4-hydroxyphenylglycol glucuronide, Flurbiprofen glucuronide, Secogalioside, Isoyatein, 9,11-Tetradecadienal, 4-phenylbutanic acid-O-sulfate, Forsythoside B, Apigenin 7-glucuronide, Betanidin 5-[E-feruloyl-(->5)-apiosyl-(1->2)-glucoside]. These significant metabolites belong to the following chemical classes: iridoid glycosides, flavonoids, lipids, oxylipin glycosides, phenolic conjugates, triterpenoid glycosides, and coumarin derivatives, with iridoid glycosides comprising the most significant proportion. Moreover, 3-O-Methylkaempferol, DHAP(6:0), Isoyatein, and 4-phenylbutanic acid-O-sulfate are the molecules that exhibit favorable profiles in terms of drug likeness compared to other molecules of the top 15 signature metabolites, and they are all up-regulated.

The metabolites of 3-O-Methylkaempferol, a naturally occurring flavonol found in several medicinal plants, exhibit documented antiviral, anti-inflammatory, and antioxidant activities, including inhibition of influenza virus replication and modulation of host immune responses [58,59]. Dihydroxyacetone phosphate derivatives, for instance DHAP (6:0), function primarily as intermediates in lipid metabolism and serve as precursors for lysophosphatidic acids and ether lipids, which can modulate cellular signaling [60,61]. Isoyatein, an isoflavone derivative, can be classified within the broader class of flavonoids, which are recognized for potential antiviral, antioxidant, and anti-inflammatory properties [62,63]. The derived metabolite 4-phenylbutanic acid-O-sulfate, which arises from sulfation of 4-phenylbutyric acid, is known for its histone deacetylase and chemical chaperone inhibition, and it is used for urea cycle disorders, with established roles in alleviation of endoplasmic reticulum stress [64,65].

Some of these significant metabolites have been studied as antiviral agents with the focus of specific SARS-CoV-2 computational or experimental research. In silico studies have identified the flavonoid derivatives 3-O-Methylkaempferol together with Apigenin 7-glucuronide as high-affinity ligands with the potential to inhibit the SARS-CoV-2 main protease (Mpro) and possibly interfere with the Spike protein’s interaction with the host ACE2 receptor [66]. Moreover, Forsythoside B is particularly noteworthy because it is a key bioactive compound found in medicinal plants such as *Forsythia suspensa*, a plant utilized in traditional Chinese medicine to treat pandemic symptoms. Its well-established broad-spectrum antiviral and strong anti-inflammatory properties, which are directly related to COVID-19 pathophysiology, justify its inclusion [67]. According to Sadowska-Bartosz and Bartosz [66], betanidin derivatives, for instance, Betanidin 5-[E-feruloyl-(->5)-apiosyl-(1->2)-glucoside], mostly function as pigments and antioxidants in plants; however, they have been less explored in antiviral studies. The other compounds, such as 3-Methoxy-4-hydroxyphenylglycol glucuronide, 4-phenylbutanic acid-O-sulfate, 7-Epi-12-hydroxyjasmonic acid glucoside, Flurbiprofen glucuronide, Secogalioside, and DHAP(6:0), have not been directly linked to SARS-CoV-2 studies.

The high levels of amino acids, fatty acids, and choline-related compounds from plant *L. javanica* and *A. calamus*, on the other hand, exhibited higher levels of acetylcarnitine, nucleosides (thymidine, deoxyguanosine), and unsaturated fatty acids. In negative ionization mode, more polar and acidic metabolites were detected, suggesting a distinct profile. Most tricarboxylic acid (TCA) cycle intermediates, as well as plant secondary metabolites, were detected in the plant *L. javanica*. These metabolites are frequently associated with energy metabolism and antioxidant defense, indicating substantial physiological adaptability and potential therapeutic efficacy [68,69]. In contrast, *A. calamus* is known to have higher amounts of glutathione disulfide, 5-oxoproline, hydroxybenzoic acid, and deoxycholic acid, all of which are involved in detoxification, redox control, and bile acid metabolism [70]. These data suggest that *A. calamus* may play a greater role in the regulation of oxidative stress and in liver-related medicinal applications. The presence of distinct metabolites in these two plants suggests differences in metabolic roles and potential therapeutic activities between the two species. The inherent complexity of medicinal plant matrices, shaped by genetic variation, environmental conditions, and processing methods, necessitates the use of highly sensitive and robust analytical tools [71,72,73].

Given the complexity of these plant matrices and the sensitivity of metabolites to environmental factors, genetics, and processing methods, data-rich, robust analytics, such as dual-polarity UPLC-MS/MS, remain crucial for reliable pharmacognosy. Limitations and subsequent phases include targeted MS/MS against authentic standards and NMR analysis of the key chemicals, including any xenobiotic-like hits. Moreover, the association of metabolites with bioactivity and in silico drug-likeness predictions remains theoretical; their therapeutic potential is inferred rather than proven, as there is no direct in vitro or in vivo validation against specific biological targets. Moreover, it is important to acknowledge that although metabolomic profiling effectively identifies potential bioactive compounds, it does not capture the synergistic or interactive effects that may underlie overall biological activity. Future work, including bioassay-guided fractionation and activity validation, is needed to confirm the functional importance of these metabolites. This would bridge the gap between untargeted metabolomic discovery and mechanistic knowledge of bioactive compound function.

Nonetheless, to our knowledge, the present study fills a critical analytical gap by applying dual-polarity UPLC-MS/MS in a phytochemical investigation of South African medicinal plants; no prior study using this strategy has been previously documented in the literature for these florae. Therefore, capturing metabolite subsets in both ionization modes improves chemical coverage and encourages future efforts in the identification of novel bioactive compounds from these species. Collectively, these findings prove that a combination of dual-mode metabolomics with multivariate prioritization enables mechanistically informed interpretation of complex plant metabolomes, moving beyond descriptive profiling toward hypothesis-driven identification of bioactive chemical signatures.

## 4. Materials and Methods

### 4.1. Selection, Collection, and Identification

The roots and leaves of *Acorus calamus* L. and *Lippia javanica* (Burm.f.) Spreng, respectively, were collected from Hartbeespoort (25.7236° S, 27.9653° E) in the North-West province of South Africa. Roots and leaves were selected as the primary medicinally used organs for each species, and organ-matched sampling was applied to enable meaningful comparative metabolomic analysis in line with ethnopharmacological practice [3,4,74]. This organ-matched sampling strategy follows metabolomics reporting recommendations that emphasize control of tissue-specific variability in comparative chemical analyses [74]. The plant collection permit was issued by the Department of Agriculture and Rural Development-Nature Conservation, South Africa (Permit No: CF6-0234; Permit Holder: Prof. Mkolo Nqobile Monate). The identity of the plants, namely *L. javanica* and *A. calamus*, was confirmed by taxonomists at the National Herbarium, where sample specimens were deposited, and voucher specimens assigned (NR 904 and NR 905, respectively).

### 4.2. In Vitro Papain-Like Protease (PL^pro^) Inhibition Assays

The selected plant extracts of *A. calamus* and *L. javanica* were evaluated for antiviral relevance using an in vitro SARS-CoV-2 papain-like protease (PL^pro^) inhibition assay. The assays were based on fluorogenic substrate cleavage and included the reference inhibitor GRL0617. Full experimental details, including assay conditions and data processing, are provided in the Supplementary Materials (Figure S1).

### 4.3. Resazurin Cell Viability Assay

HEK293T cells from Cellonex Separation Scientific SA (Pty) Ltd., Johannesburg, South Africa were used for the resazurin cell viability assay. The cells were cultured in 10% fetal bovine serum (FBS) supplemented Dulbecco’s Modified Eagle’s Medium (DMEM) (Thermo Fisher Scientific (Gibco), Waltham, MA, USA). A sterile laminar flow hood washed with 70% ethanol was used to handle the cells. The cells (non-contaminated HEK293T (1.13 × 10^6^ cells/mL)) in medium were then cultured in an incubator with 5% CO_2_ at 37 °C for a period of 24 h. In addition to 20 µL of resazurin dye (TOX-8, Sigma-Aldrich, St. Louis, MO, USA), the cells were seeded at 5000 cells/well in 100 µL medium and treated with methanol extracts of *A. calamus* and *L. javanica* leaves. DMSO (0.5%) was used to prepare plant extracts at concentrations of 250, 125, 62.5, 31.25, 15.63, and 7.81 µg/mL. Using an excitation wavelength (EX) of 560 nm, the Modulus II Multifunction Plate Reader (Turner BioSystems, Sunnyvale, CA, USA) was utilized to measure the rise in resorufin fluorescence at a wavelength (EM) of 590 nm. Resorufin had an absorbance peaking at 570 nm, while resazurin’s absorbance peaked at 600 nm.

### 4.4. Metabolomics: Establishment of the Metabolites

#### 4.4.1. Instruments and Reagents

Ultimate 3000LC combined with Q Exactive MS (Thermo Fisher Scientific, Bremen, Germany), Temp functional Centrifugation (Eppendorf, Hamburg, Germany), ACQUITY UPLC HSS T3 (100 mm × 2.1 mm × 1.8 μm) were used. LC–MS-grade acetonitrile, methanol, formic acid, and DL-o-Chlorophenylalanine were purchased from Merck (Darmstadt, Germany).

#### 4.4.2. Sample Preparation

**Figure 9:**
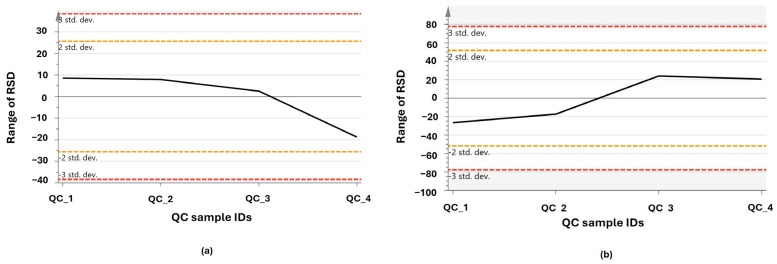
Quality control (QC) plot samples: (**a**) Negative ionization mode; (**b**) Positive ionization mode. Showing the X axis indicates the quality control sample identifiers (QC sample IDs), and the Y axis indicates the range of relative standard deviation (RSD). The black line represents the cumulative QC-based RSD deviation across sequential QC injections, signifying temporal stability of the LC–MS system. Each QC sample was injected in triplicate.

The samples of *A. calamus* and *L. javanica* were lyophilized to dryness and subsequently crushed in a 5-mL homogenizing tube at 30 Hz with four 5-mm metal balls in an MM 400 mill mixer to a fine powder. Fifty milligrams of each sample was precisely weighed into a tube, and 800 μL of 80% methanol was added. Then, the samples were vortexed for 30 s, followed by sonication for 30 min at 4 °C. All samples were kept at −20 °C for 1 h, then centrifuged at 12,000 rpm at 4 °C for 15 min. Finally, 200 μL of supernatant and 5 μL of DL-o-Chlorophenylalanine (0.14 mg/mL) were added as an internal standard to each sample before LC-MS analysis. The exact amount of extract from each sample was determined and combined with the QC samples to evaluate the methodology. QC samples were used to demonstrate the stability of the LC-MS system. The ion features of the QC samples were used to calculate the relative standard deviation (RSD). The % RSD distribution is presented in Figure 9; the overwhelming majority of RSD values were less than 30%. Therefore, this indicates that the analysis procedure was robust and was subsequently applied to subsequent sample analysis. QC samples and internal standards were used for normalization and quality control during preprocessing but were excluded from PCA visualization to prevent clustering bias [25].

#### 4.4.3. Metabolomics Analysis of Plant Samples Using UPLC-MS/MS

Separation was performed using an Ultimate 3000 LC system coupled to a Q Exactive MS (Thermo) and screened by ESI-MS. The LC system comprises an ACQUITY UPLC HSS T3 (100 mm × 2.1 mm, 1.8 μm) with an Ultimate 3000 LC. The mobile phase was composed of solvent A (0.05% formic acid water) and solvent B (acetonitrile) with a gradient elution (0–1 min, 95% A, 1–12 min, 5–95% A, 12–13.5 min, 5% A, 13.5–13.6 min, 5–95% A, 13.6–16 min, 95% A). The mobile-phase flow rate was 0.3 mL·min^−1^. The column temperature was maintained at 40 °C, and the sample manager temperature was set at 4 °C. Samples were analyzed in both negative- and positive-ionization modes to compare secondary metabolites in the extracts. Mass spectrometry parameters in electrospray ionization (ESI), ESI positive (ESI+) and ESI negative mode (ESI−) modes are listed as follows: ESI+: The heater (desolvation) temperature was set to 300 °C, the sheath gas flow rate to 45 arbitrary units (arb), the auxiliary gas flow rate to 15 arb, and the sweep gas flow rate to 1 arb. The spray voltage was maintained at 3.0 kV, the capillary temperature at 350 °C, and the S-Lens RF level at 30%.

ESI−: The heater (desolvation) temperature was set to 300 °C, the sheath gas flow rate to 45 arbitrary units (arb), the auxiliary gas flow rate to 15 arb, and the sweep gas flow rate to 1 arb. The spray voltage was maintained at 3.2 kV, the capillary temperature at 350 °C, and the S-Lens RF level at 60%.

#### 4.4.4. Identification of Metabolites

The chemical structures of important metabolites were putatively identified according to online databases such as the Human Metabolome Database, www.hmdb.ca (accessed on 19 August 2024), Chemspider, www.chemspider.com (accessed on 19 August 2024), and Mass Bank, www.massbank.jp (accessed on 19 August 2024), using the data of accurate masses and MS/MS fragments. Moreover, each candidate identification was manually confirmed by evaluation of retention time, isotope pattern, and MS/MS fragmentation spectra, and afterward cross-validation across the three databases to confirm spectral consistency. Duplicating entries detected in both ESI− and ESI+ modes were cross-checked for spectral and chromatographic resemblance, and, where necessary, they were merged into a single consensus feature. Redundant ions detected in both ESI− and ESI+ modes were also merged by employing a systematic selection approach that ranked higher signal intensity, lower background noise or matrix interference, and lower %RSD across technical replicates. Moreover, adduct formation was accounted for during metabolite annotation by utilizing manual adduct assignment, retention-time coherence, strict mass accuracy criteria (<5 ppm), and cross-mode validation to avoid duplication or erroneous assignments across ESI modes.

### 4.5. In Silico Pharmacokinetic Prediction and Drug-Likeness Evaluation

The Swiss ADME database, http://www.swissadme.ch/index.php (accessed on 12 June 2025), was utilized to predict the water solubility, lipophilicity, pharmacokinetic profiles, drug-likeness, and medicinal chemistry of the top 15 signature metabolites [59].

### 4.6. Statistical Analysis

GraphPad Prism version 8.2.0 (GraphPad Software, Inc., San Diego, CA, USA) was utilized for statistical analysis (means ± SD and ANOVA *p*-values) of the resazurin cell viability assay. The raw data used for metabolomics analysis were acquired and aligned using Compound Discover (3.0; Thermo) based on *m*/*z* values and retention times. Ions from both ESI− and ESI+ were merged and imported into the SIMCA-P program (version 14.1) for multivariate analysis. Principal Components Analysis (PCA) was first used as an unsupervised method for data visualization and outlier identification. Supervised regression modeling was then performed on the dataset by using Partial Least Squares Discriminant Analysis (PLS-DA) or Orthogonal Partial Least Squares Discriminant Analysis (OPLS-DA) to identify the potential biomarkers. The biomarkers were filtered and confirmed by combining the results of the VIP values (VIP > 1.5), *t*-test (*p* < 0.05), and a fold change (FC > 2.0). Multiple comparison correction to reduce false positives (FDR-corrected *p*-values (q-values) < 0.05) was performed using Benjamini–Hochberg false discovery rate (FDR). The quality of the fitting model was explained by R^2^ and Q^2^ values. R^2^ displays the variance explained in the model and indicates the quality of the fit. Q^2^ displays the variance in the data, indicating the model’s predictability.

## 5. Conclusions

This study reveals the value of dual-polarity UPLC-MS/MS metabolomics as an analytical framework for comparative phytochemical profiling of ethnomedicinal plants. By integrating ESI+ and ESI− ionization modes, we achieved expanded metabolome coverage and improved confidence in metabolite annotation, enabling clear discrimination between *L. javanica* and *A. calamus* despite their botanical divergence.

Univariate and multivariate analyses revealed different chemotypic signatures dominated by iridoid glycosides, flavonoids, and jasmonate-derived oxylipins, several of which exhibited favorable drug-likeness properties. These findings position dual-mode metabolomics as a powerful pre-mechanistic prioritization approach that bridges chemical profiling with early functional validation, as further supported by PL^pro^ inhibition assays, without overstretching claims of therapeutic efficacy.

Although direct antiviral mechanisms remain to be established at the compound level, incorporating metabolomic prioritization, cytotoxicity screening, and targeted antiviral assays provides a rational pathway for bioassay-guided fractionation and mechanistic studies. Collectively, this study advances metabolomics-driven natural product discovery and underscores South African medicinal plants as valued sources of chemically and biologically significant metabolites for future antiviral research. Flavonols, scaffolds of 3-O-methylkaempferol, Pollenin A, and jasmonate-pathway derivatives of 7-epi-12-hydroxyjasmonic acid were among the significant metabolites that demonstrated favorable drug-likeness. With targeted MS/MS and absolute quantification as the subsequent orthogonal points.

## Figures and Tables

**Table 1:** Identified top-fifteen significant compounds with the highest VIP score of *L. javanica* and *A. calamus* (ESI+ mode).

Metabolites	HMDB ID	Formula	Class	*m*/*z*	RT [min]	Log_2_ (FC)	*t*-Test	FDR Log_10_ (*p*-adj)	VIP
2′,3′-Didehydro-2′,3′-dideoxycytidine	HMDB0245545	C_9_H_11_N_3_O_3_	PN	210.08839	4.434	−8.766	1.281 × 10^−5^	4.893	2.093
7-Epi-12-hydroxyjasmonic acid	HMDB0303749	C_12_H_18_O_4_	FA	227.12729	3.942	9.757	2.825 × 10^−8^	7.549	2.219
Isopropyl beta-glucoside	HMDB0032705	C_9_H_18_O_6_	OO	245.10156	3.5	10.833	3.517 × 10^−5^	4.454	2.331
Pollenin A	HMDB0303704	C_15_H_10_O_7_	FV	303.0493	4.254	8.769	2.164 × 10^−7^	6.665	2.127
3′-C-Ethynylcytidine	HMDB0252093	C_11_H_13_N_3_O_5_	PN	309.11736	3.174	8.752	3.191 × 10^−8^	7.496	2.107
6b-Hydroxymethandienone	HMDB0005832	C_20_H_28_O_3_	AD	339.19221	10.138	10.735	2.655 × 10^−6^	5.576	2.318
2-trans-O-Feruloylglucaric acid	HMDB0302546	C_16_H_18_O_11_	OO	387.09133	3.606	9.918	9.379 × 10^−6^	5.028	2.227
DG(20:4-2OH/0:0/2:0)	HMDB0297002	C_25_H_40_O_7_	DG	475.26816	8.79	−8.772	1.311 × 10^−5^	4.882	2.095
Quercetin 3-O-glucuronide	HMDB0029212	C_21_H_18_O_13_	FV	479.08117	4.241	9.420	6.675 × 10^−8^	7.176	2.186
Persiconin	HMDB0037482	C_23_H_26_O_11_	FV	479.15387	4.285	8.488	1.030 × 10^−6^	5.987	2.064
Apigenin 7-O-diglucuronide	HMDB0301685	C_27_H_26_O_17_	FV	623.1222	4.027	9.063	3.859 × 10^−7^	6.414	2.131
Hesperidin methylchalcone	HMDB0253112	C_29_H_36_O_15_	FV	625.21058	4.557	9.090	1.041 × 10^−8^	7.982	2.140
Tetramethylquercetin 3-rutinoside	HMDB0039337	C_31_H_38_O_16_	FV	708.24685	4.052	11.679	1.567 × 10^−6^	5.805	2.419
Hetastarch	HMDB0253113	C_29_H_52_O_21_	PS	759.2936	4.045	8.837	2.091 × 10^−5^	4.680	2.135
Leonoside A	HMDB0040342	C_35_H_46_O_19_	OS	788.2936	4.557	9.612	1.246 × 10^−5^	4.904	2.197

Key: Pyrimidine nucleosides (PN); Fatty Acyls (FA); Organooxygen (OO); Flavonoid (FV); Androgens and derivatives (AD); Diacylglycerols (DG); Polysaccharides (PS); Oligosaccharides (OS).

**Table 2:** Identified top-fifteen significant compounds with the highest VIP score of *L. javanica* and *A. calamus* (ESI− mode).

Compound Name	HMDB_ID	Formula	Class	*m*/*z*	RT [min]	Log_2_ (FC)	*t*-Test	FDR Log_10_ (*p*-adj)	VIP
9,11-Tetradecadienal	CSID24841995	C_13_H_22_O	UFA	207.1748	8.864	−11.453123	1.6895 × 10^−6^	5.772	1.9290707
3-O-Methylkaempferol	HMDB0302564	C_16_H_12_O_6_	FV	299.0559	6.743	10.790121	4.73057 × 10^−6^	5.325	1.8594932
DHAP(6:0)	HMDB0011679	C_9_H_17_O_7_P	FA	313.0717	7.551	11.157672	5.49481 × 10^−6^	5.260	1.8948333
7-Epi-12-hydroxyjasmonic acid glucoside	HMDB0040706	C_18_H_28_O_9_	FA	387.1656	3.925	11.510354	1.40757 × 10^−6^	5.852	1.9225042
Oleoside	HMDB0303576	C_16_H_22_O_11_	PL	389.1084	3.55	11.166624	9.78139 × 10^−8^	7.010	1.9118317
3-Methoxy-4-hydroxyphenylglycol glucuronide	HMDB0000496	C_15_H_20_O_10_	P	405.1037	3.642	10.950677	4.06598 × 10^−5^	4.391	1.9175946
Flurbiprofen glucuronide	HMDB0060918	C_21_H_21_FO_8_	OG	419.1184	4.129	14.007274	6.21963 × 10^−5^	4.206	2.1283598
Secogalioside	CSID391591	C_17_H_24_O_12_	PL	419.1196	3.453	11.16158	9.03621 × 10^−7^	6.044	1.8856703
Isoyatein	HMDB0033258	C_22_H_24_O_7_	FL	435.1139	3.58	13.134569	2.96326 × 10^−5^	4.528	2.0475777
4-phenylbutanic acid-O-sulfate	HMDB0059983	C_10_H_12_O_5_S	BSD	487.0756	2.468	11.623369	2.86636 × 10^−5^	4.543	1.9271363
6-beta-Glucopyranosyl-4′,5-dihydroxy-3′,7-dimethoxyflavone	HMDB0037568	C_23_H_24_O_11_	FV	511.109	2.88	−11.981789	0.000194167	3.712	1.9489596
Theasapogenol E	HMDB0034518	C_30_H_48_O_6_	TP	549.3433	11.628	−11.223913	0.000289929	3.538	1.8939477
Forsythoside B	HMDB0252461	C_34_H_44_O_19_	OS	755.2405	4.271	13.298272	3.36428 × 10^−9^	8.473	2.0628614
Apigenin 7-glucuronide	HMDB0240480	C_21_H_18_O_11_	FV	891.1627	5.026	11.284524	1.10247 × 10^−7^	6.958	1.9010577
Betanidin 5-[E-feruloyl-(→5)-apiosyl-(1→2)-glucoside]	HMDB0037052	C_39_H_42_N_2_O_20_	ICA	903.2567	4.743	11.98583	1.17592 × 10^−6^	5.930	1.9577122

Key: Unsaturated fatty aldehyde (UFA); Flavonoid (FV); Fatty Acyls (FA); Prenol lipids (PL); Phenols (P); O-glucuronides (OG); Furanoid lignans (FL); Benzene and substituted derivatives (BSD); Triterpenoids (TP); Oligosaccharides (OS); Indolecarboxylic acids (ICA).

**Table 3:** Parameters for PCA, PLS-DA, and OPLS-DA models for *L. javanica* and *A. calamus* metabolite groups.

Parameter	Model	Number of Model Dimensions	Total Samples Number (N)	R^2^X (cum)	R^2^Y (cum)	Q^2^ (cum)
Negative mode	PCA-X	2	12	0.86		0.786
	PLS-DA	2	12	0.857	1	1
	OPLS-DA	1 + 1 + 0	12	0.857	1	0.999
Positive mode	PCA-X	2	12	0.799		0.697
	PLS-DA	2	12	0.793	1	0.999
	OPLS-DA	1 + 1 + 0	12	0.793	1	0.999

## Data Availability

Data is included in the article or Supplementary Materials; further inquiries can be directed to the corresponding author.

## Supplementary Materials

The following supporting information can be downloaded at https://www.mdpi.com/article/10.3390/plants15020232/s1. Figure S1: In vitro inhibition of SARS-CoV-2 papain-like protease (PLᵖʳᵒ) by selected extracts of *A. calamus* and *L. javanica* and the reference inhibitor GRL0617. Table S1: Signifies comprehensive chemical profiled metabolites and other details from *L. javanica* and *A. calamus* (ESI + and ESI−). Table S2: Pharmacokinetic properties and drug-likeness evaluation for positive and negative ionization modes.
