# Supplementary material for: Identifying Key Metabolites in South African Medicinal Plants Using Dual Electrospray Ionization Metabolomics
Source: Plants (Basel). 2026 Jan 12;15(2):232. doi: 10.3390/plants15020232 (PMC12844898; doi:10.3390/plants15020232)
Supplement: Supplementary file 1 [file plants-15-00232-s001.zip › Figure S1 supplimentary material-R1.pdf]

Supplementary materials

In Vitro Studies

1.1. Reagents, Assay Kits, and Compounds

Two complementary assay systems were used to evaluate SARS-CoV-2 papain-like protease (PLpro) function: the Protease Activity Kit (BPS Bioscience, San Diego, California, USA) and the Deubiquitinase Activity Kit (BPS Bioscience, San Diego, California, USA). Both kits were provided in a 96-well plate format and included recombinant PL^{pro} (SARS-CoV-2, His-tag), fluorogenic substrates, assay buffer, dithiothreitol (DTT), and the non-covalent PL^{pro} inhibitor GRL0617 as a positive control.

1.2. Preparation of Assay Buffer and Enzyme and Samples Preparation

For each assay, a stock solution of 0.5 M dithiothreitol (DTT) was diluted in the supplied assay buffer to a final concentration of 1 mM. Recombinant PL^{pro} was thawed on ice, briefly centrifuged, and aliquoted into single-use portions to minimize freeze–thaw degradation. For protease activity assays, PL^{pro} was diluted in buffer to a working concentration of 0.3-0.5 ng/μL, corresponding to 9-15 ng per reaction, but for deubiquitinase assays, the working concentration was adjusted to 0.7-1 ng/μL (21-30 ng per reaction).

Methanolic plant extracts were dissolved in DMSO, and the final DMSO concentration in the assay did not exceed 1% (v/v). Stock solutions were prepared at 100× the desired working concentration and then diluted 20-fold in assay buffer containing 1 mM DTT to achieve final extract concentrations of 1, 3, 10, 30, 100, and 300 μg/mL. The reference inhibitor GRL0617 served as a positive control (10 mM stock solution in DMSO) and was further diluted in 500 μM assay buffer to yield final concentrations of 0.3, 1, 3, 10, 30, and 100 μM. Blank wells containing assay buffer lacking enzyme were added to account for background fluorescence.

1.2.1. Protease Activity and Deubiquitinase Activity Assays

Protease activity was assessed by adding 30 μL of diluted PL^{pro} solution to 96-well plates in triplicate, except for blank controls, which received only assay buffer. To test the sample wells, 10 μL of the prepared extract and 10 μL of the GRL0617 solution were added. After a 30 min pre-incubation at 37 °C, 10 μL of a 25 μM fluorogenic substrate was added to initiate the reaction. Plates were sealed and incubated at 37 °C for 45-60 min. Fluorescence was measured at excitation (λ_exc 360 nm) and emission wavelengths (λ_em 460 nm) using a multimode microplate reader.

Deubiquitinase activity was evaluated using the protease activity assay, with minor modifications. After PL^{pro} and test solutions were added as described in the foregoing section, reactions were initiated with 10 μL of a diluted ubiquitinated fluorogenic substrate at a final concentration of 250 nM. Plates were incubated for 45-60 min at 37 °C. Fluorescence was also recorded under the same excitation or emission wavelengths (λ_exc 360 λ_em 460 nm). Blank values were subtracted from all readings before analysis. Dose–response curves and IC_{50} values were generated using GraphPad Prism 10 (GraphPad Software, La Jolla, CA, USA).

Results

The results support the metabolomics-guided prioritization strategy employed in the main manuscript by providing functional validation of selected plant extracts of *A. calamus* and *L. javanica* against a validated antiviral target (Figure 1S). *L. javanica* (IC_{50} I9.15 μg/mL; IC_{50} 12.3 μg/mL) and *A. calamus* (IC_{50} 10.2 μg/mL; IC_{50} 14.1 μg/mL) extracts and the benchmark inhibitor GRL0617 (IC_{50} 3.4 μM; IC_{50} 4.6 μM) exhibited dose-dependent inhibition of SARS-CoV-2 PL^{pro} protease and deubiquitinase activities, respectively (Figure 1S).

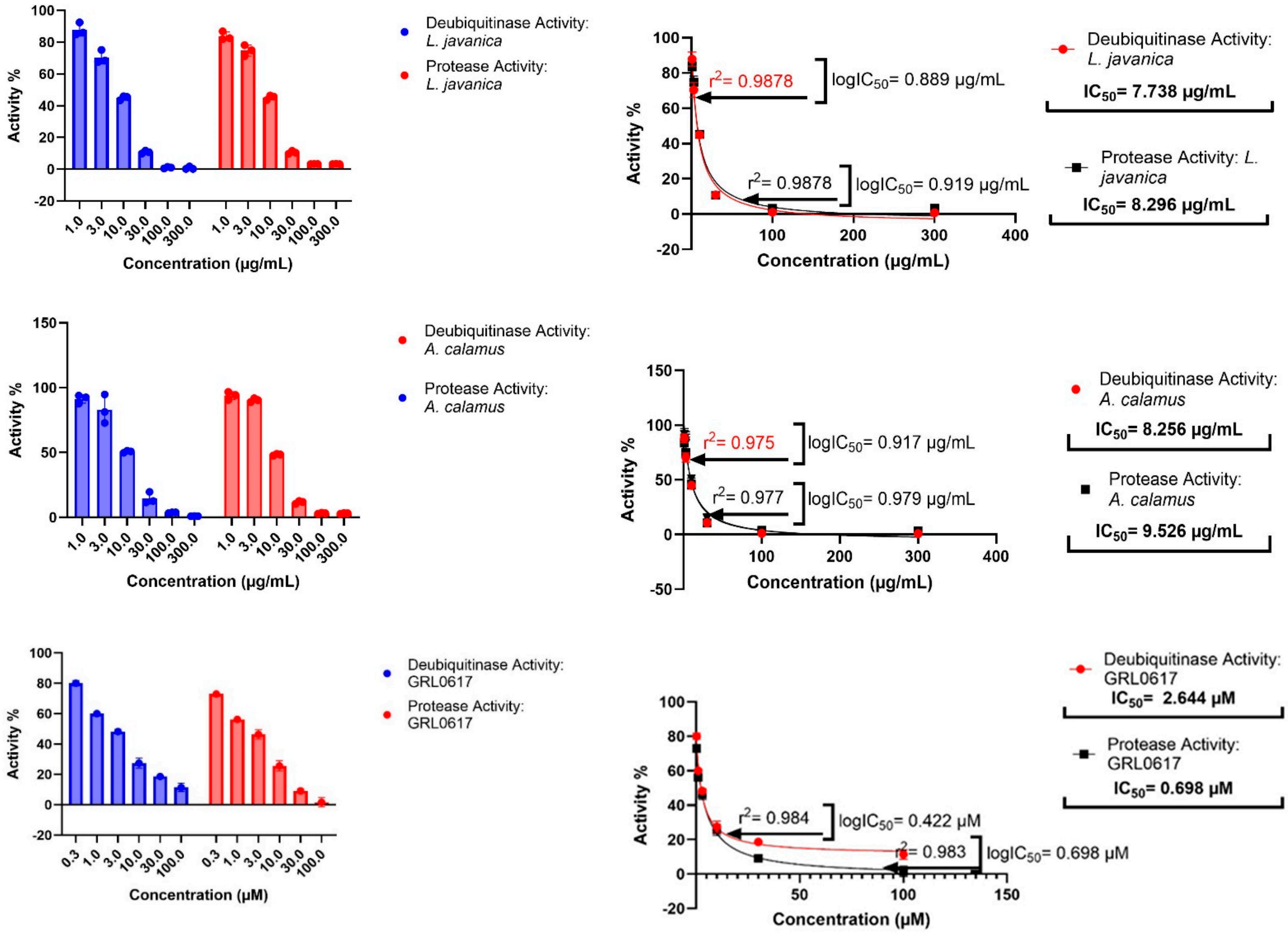

Figure S1: In vitro inhibition of SARS-CoV-2 papain-like protease (PL^{pro}) by selected extracts of *A. calamus* and *L. javanica* and the reference inhibitor GRL0617. The results support the metabolomics-guided prioritization strategy employed in the main manuscript by providing functional validation of selected extracts against a validated antiviral target.
